# Social alienation and cognitive function among older adults: the mediating roles of ruminative thinking and self-neglect

**DOI:** 10.3389/fpubh.2026.1820738

**Published:** 2026-06-11

**Authors:** Wen-Ting Xia, Yi-Ran Yue, Yue Zhang, Jia Tao, Dan Su

**Affiliations:** 1 School of Nursing, Anhui Medical University, Hefei, China; 2 The First Affiliated Hospital of Anhui Medical University, Hefei, China; 3 The Taikang Health and Wellness Industry Research Institute, Anhui Medical University, Hefei, China

**Keywords:** cognitive function, mediating associations, older adults, ruminative thinking, self-neglect, social alienation, stress process model

## Abstract

**Background:**

Cognitive decline has become a major public health concern in the context of global population aging. Social alienation has been identified as a significant modifiable factor associated with cognitive impairment. Although previous studies have suggested that depression mediates the association between social alienation and cognitive function, depression is a multidimensional construct. The specific social-psychological-behavioral associations underlying this relationship remain unclear. This study aimed to examine the mediating associations of ruminative thinking and self-neglect in the relationship between social alienation and cognitive function, based on the stress process model, and to conduct an exploratory reversed-model analysis involving self-neglect and cognitive function.

**Methods:**

A cross-sectional study design was employed. From May to November 2025, 340 older adults were recruited in Anhui Province, China. The Generalized Alienation Scale, Ruminative Responses Scale, The Scale of the Elderly Self-Neglect, and Montreal Cognitive Assessment were used. Data analysis was conducted using SPSS and AMOS. Structural equation modeling (SEM) was conducted, and the mediating associations were examined using the bootstrap method.

**Results:**

Cognitive function was negatively correlated with social alienation (*r* = −0.749, *p* < 0.01), ruminative thinking (*r* = −0.724, *p* < 0.01), and self-neglect (*r* = −0.742, *p* < 0.01). The estimated total association between social alienation and cognitive function included a direct association (β = −0.364, 95% CI = −0.606,−0.178) and an indirect association (β = −0.456, 95% CI = −0.643,−0.269). The sequential indirect association involving ruminative thinking and self-neglect was statistically significant (β = −0.091, 95% CI = −0.232,−0.017), accounting for 11.10% of the estimated total association. The exploratory reversed model showed comparable fit, with a significant reversed sequential indirect association (β = 0.120, 95% CI = 0.023, 0.283).

**Conclusion:**

This study identified a potential socio-psychological-behavioral pattern linking social alienation, ruminative thinking, self-neglect, and cognitive function in older adults. The reversed model suggested that the temporal ordering between self-neglect and cognitive function could not be definitively established. These findings suggest that public health strategies aimed at promoting cognitive health may consider integrating social participation enhancement, psychological support, behavioral guidance, and cognitive risk screening.

## 1 Introduction

With rapid global population aging, age-related cognitive decline has become an important public health challenge. The World Health Organization estimates that approximately 55 million people worldwide are living with cognitive impairment, and this number continues to rise (1). Cognitive impairment is one of the common problems faced by older adults, typically progressing from subjective cognitive decline to mild cognitive impairment (MCI), and some individuals may further develop Alzheimer's disease (AD). It is mainly characterized by deficits in executive function, visuospatial ability, attention, and memory (2, 3). Declining cognitive function not only affects independent living and social participation in older adults, but also increases the caregiving burden on families and society (4, 5). However, not all individuals with MCI will progress to AD. Active lifestyle factors, such as healthy sleep, diet, and chronic disease management, have been associated with slower cognitive decline. Therefore, identifying modifiable factors associated with cognitive decline and clarifying their underlying associations is important for promoting healthy aging.

In recent years, psychosocial factors have attracted increasing attention in research on cognitive health in older adults. In nursing diagnosis, social alienation refers to a subjective feeling of being isolated, marginalized, and lacking a sense of belonging in social interactions, and it is regarded as a common adverse psychosocial state in later life (6, 7). Previous studies have shown that social alienation is significantly associated with lower levels of cognitive function and is considered one of the important modifiable factors related to cognitive impairment. More importantly, social alienation is not only linked to cognitive decline but is also considered a modifiable and intervention-targetable factor (8–11). Some studies have also suggested that depression may mediate the relationship between social alienation and cognitive function (12). However, depression is a complex emotional disorder that involves cognitive, emotional, and behavioral aspects, so it cannot fully explain the specific psychological and behavioral links between social alienation and cognitive function (13). Therefore, further research is needed to examine more specific mediating factors in this relationship.

Pearlin's stress process model provides a useful theoretical framework for understanding how psychosocial factors are associated with health outcomes. According to this model, chronic stressors may be associated with health through individuals' cognitive responses and behavioral adaptation processes (14). In this study, social alienation is conceptualized as a chronic psychosocial stressor. Within this theoretical framework, chronic stressors may co-occur with maladaptive cognitive responses and behavioral dysregulation, which may be statistically associated with poorer cognitive function.

At the cognitive response level, ruminative thinking is a maladaptive pattern of thinking that involves repeatedly focusing on negative emotions and their causes (15). Previous studies have suggested that ruminative thinking is associated with greater cognitive resource demands, emotional distress, and poorer executive control (16, 17). For older adults with social alienation, limited social support and reduced social interaction may be associated with greater repetitive negative thinking. Therefore, ruminative thinking may be an important psychological factor linking social alienation to cognitive function.

At the behavioral level, self-neglect refers to the situation where older adults fail to meet their basic living needs either intentionally or unintentionally (18). Although self-neglect in the advanced stage of Alzheimer's disease and related behavioral dysfunctions are often observed alongside impairments in daily living abilities and emotional control that may accompany neurodegenerative progression, in the early stage of cognitive decline, these manifestations may be closely related to self-regulatory difficulties in the context of psychological and social stress. Previous studies have shown that this disorder is not only associated with poorer physical health but also with lower cognitive function (19, 20).

**Figure 1:**
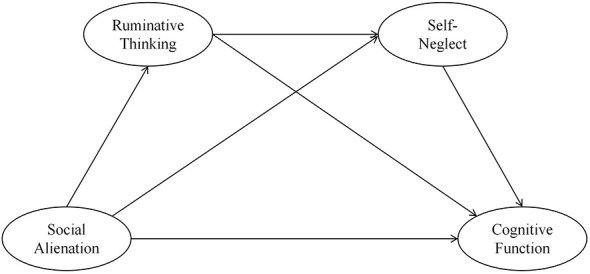
Theory-informed hypothesized mediation model.

In summary, social alienation, ruminative thinking, and self-neglect are all closely related to cognitive function in older adults. However, the specific relationships among these variables and their possible mediating associations are still not fully clear. Based on the stress process model, this study viewed social alienation as a chronic psychosocial stressor, ruminative thinking as a maladaptive cognitive response, self-neglect as a manifestation of behavioral dysregulation, and cognitive function as the health outcome. On this basis, a theory-informed mediation model was developed to examine how social alienation may relate to cognitive function through ruminative thinking and self-neglect. Given the close conceptual relationship between self-neglect and cognitive function, an exploratory reversed model was also tested to assess whether the ordering between these two constructs was uniquely supported by the data. Based on this framework, the following hypotheses were proposed (Figure 1):

H1: Social alienation will be significantly associated with cognitive function.

H2: A statistically significant indirect association between social alienation and cognitive function will be observed through ruminative thinking.

H3: A statistically significant indirect association between social alienation and cognitive function will be observed through self-neglect.

H4: A statistically significant sequential indirect association between social alienation and cognitive function will be observed through ruminative thinking and self-neglect.

In addition to the hypothesized model, the exploratory reversed model was tested as a sensitivity analysis rather than as a predefined hypothesis.

## 2 Methods

### 2.1 Design

This is a cross-sectional study on the cognitive function of older adults, following the STROBE guidelines for strengthening observational epidemiological reports (21).

### 2.2 Sample size

To ensure adequate sample size, this study adopted the method recommended by MacCallum et al. (22) and conducted *a priori* statistical power analysis based on the root mean square error of approximation (RMSEA). Given the degrees of freedom (*df* = 218) of the final structural model, under the condition of setting the significance level α = 0.05 and the expected statistical power of 0.80, the minimum sample size required to test the hypotheses of good fit (RMSEA = 0.05) and moderate fit (RMSEA = 0.08) was 80. The actual effective sample size of this study was 340, which far exceeded this minimum standard. Therefore, the sample size was sufficient to ensure adequate statistical power for the parameter estimation and model testing of the structural equation model.

### 2.3 Participants

To enhance sample representativeness, this study recruited participants from both community centers and hospital settings, aiming to cover a diverse group of older adults with different health conditions. Therefore, from May to November 2025, convenience sampling was used to recruit participants from two tertiary Grade A hospitals and two community centers in Anhui Province, China. In the hospital settings, participants were mainly recruited from outpatient clinics and inpatient wards of the Department of Geriatric Medicine, with a smaller proportion recruited from neurology outpatient clinics. In the community settings, participants were recruited through routine older adult health management, health examinations, and community-based health education activities.

Before formal enrollment, all potential participants underwent an eligibility screening procedure conducted by uniformly trained researchers. The screening included a brief assessment of demographic information, communication ability, medical history, and previous diagnoses of major neurological or psychiatric disorders. For participants recruited from hospital settings, available medical records and clinical information were reviewed when accessible. For participants recruited from community settings, self-reported medical history was supplemented, when necessary, by information from family members or primary caregivers. Particular attention was paid to whether participants had a previous diagnosis of dementia, severe neurocognitive disorder, acute cerebrovascular disease, Parkinson's disease, or other neurological conditions that could substantially affect communication or assessment completion.

Participants were required to complete a test on social alienation, ruminative thinking, self-neglect, and cognitive function. Inclusion criteria were as follows: (1) age ≥ 60 years old; (2) having basic communication ability and the ability to understand and cooperate with the assessment of the scale; (3) voluntarily participating in this study and signing the informed consent form. Exclusion criteria included: (1) severe visual or auditory impairments or aphasia, which prevent effective communication; (2) severe mental disorders such as schizophrenia that make it impossible to cooperate with the investigation; (3) severe physical or central nervous system diseases in the acute stage, such as acute stroke or acute cerebral hemorrhage within the previous 2 weeks; (4) previously diagnosed dementia, severe neurocognitive disorder, Parkinson's disease, stroke with severe sequelae, or other neurological diseases that substantially affected communication or assessment completion; (5) being in the terminal stage of the disease or having completely lost the ability to express independently; (6) participated in similar studies within the past 3months.

Participants with lower cognitive performance were not excluded solely on the basis of their cognitive assessment results. They were eligible for inclusion if they had no previous diagnosis of dementia or severe neurocognitive disorder, were able to understand the study procedures, and could communicate their own responses during the assessment.

### 2.4 Tools

#### 2.4.1 General information questionnaire

This questionnaire was developed based on a literature review and the study objectives. It includes information such as gender, age, marital status, educational background, occupation, residential area, living situation, frequency of social activities participation, and physical exercise situation.

#### 2.4.2 Generalized alienation scale (GAS)

This scale was developed by Jessor et al. and was revised and translated into Chinese by Wu Shang et al. (23), and is used to measure an individual's level of social alienation. The scale is divided into four dimensions: self-alienation (including 3 items: 9, 10, and 11), other-alienation (including 5 items: 1, 4, 7, 8, and 12), suspicion (including 4 items: 3, 6, 14, and 15), and meaninglessness (including 3 items: 2, 5, and 13). There are a total of 15 items. It uses the Likert 4-point rating method, with each item scored from “strongly disagree” to “strongly agree” as 1 to 4 points respectively. The total score ranges from 15 to 60, with a higher score indicating a higher level of social alienation. The Cronbach's α coefficient of this scale was 0.776. In this study, the test-retest reliability was 0.886.

#### 2.4.3 Ruminative responses scale (RRS)

The scale selected for this study is the revised Ruminative Responses Scale by Han Xiu (15). The Cronbach's α coefficient of this scale was 0.90, and the test-retest reliability of this study was 0.919. The content of this scale includes three dimensions: brooding (including 5, 10, 13, 15, 16, totaling 5 items), reflective pondering (7, 11, 12, 20, 21, totaling 5 items), and symptom rumination (including 1, 2, 3, 4, 6, 8, 9, 14, 17, 18, 19, 22, totaling 12 items). In total, it contains 22 main items. The scores of each item are assessed using the Likert 4-point scale, with 1–4 corresponding to never, sometimes, often, and always. The total score of the scale ranges from 22 to 88 points, with higher scores indicating more severe ruminative thinking. Scores of 66–88 indicate a high level of ruminative thinking, 44–65 indicate a medium level, and 22–43 indicate a low level.

#### 2.4.4 The scale of the elderly self-neglect (SES)

This scale was developed by Zhao Yuanyuan (24) and is mainly targeted at rural older adults. It consists of 5 dimensions (medical care (items 1, 2, 3), hygiene (items 4, 5, 6), emotion (items 7, 8, 9), safety (items 10, 11, 12), and social interaction (items 13, 14)) and 14 items. The scores for each item are assessed using the Likert 4-point scale (0–3 points). The higher the total score obtained, the more severe the degree of self-neglect. Although the early development of this scale was mainly focused on rural older adults, the core dimensions it covers (such as medical care, hygiene, safety, etc.) reflect the universal needs of older adults to maintain basic health. These needs are theoretically not limited by urban or rural backgrounds, ensuring content validity. Additionally, existing studies have verified the applicability of this scale in urban community samples, and its reliability and validity are good (25). The Cronbach's α coefficient of this scale was 0.801. In this study, the test-retest reliability was 0.876, demonstrating good reliability.

#### 2.4.5 Montreal cognitive assessment (MoCA)

This scale was developed and validated by Nasreddine et al. from Canada (26) based on clinical experience and by referring to the cognitive items and scoring of MMSE. This scale assesses the cognitive function of older adults from 8 dimensions: visuospatial and executive ability, naming, attention, calculation, language, abstraction, delayed memory, and orientation. The total score of the scale is 30 points. The higher the score, the better the cognitive function. According to the developers' guidelines, 1 point was added to the total score for participants with ≤ 12 years of education to adjust for educational background. Given that educational level is an important confounding factor in cognitive assessment of the older adult population in China, this study refers to the large-sample validation research conducted by Lu et al. (27) for the older adult population in China, and adopts the optimal cut-off point based on educational level to determine the cognitive function status: for those with primary school education or below, a total score of ≤ 19 points indicates the presence of cognitive dysfunction; for those with junior high school education or above, a total score of ≤ 24 points indicates the presence of cognitive dysfunction. The Cronbach's α coefficient of this scale was 0.818. In this study, the test-retest reliability is 0.851 and the test-retest validity is 0.919.

### 2.5 Data collection

Data were collected through face-to-face interviews. Before questionnaire administration, all potential participants from both hospital and community settings were screened according to the eligibility procedure described above. A total of 361 older adults were assessed for eligibility, of whom 16 were excluded during the preliminary screening stage because they did not meet the inclusion criteria, met the exclusion criteria, or declined to participate. Specifically, 5 participants were excluded because of a previous diagnosis of dementia or severe neurocognitive disorder, 3 because of acute or severe neurological disease, 3 because of severe communication barriers such as aphasia or severe hearing impairment, 1 because of severe psychiatric disorder, 2 because they were in the terminal stage of disease or had completely lost the ability to express independently, and 2 because they declined to participate. After eligibility screening, a total of 345 questionnaires were distributed to eligible participants.

To ensure data quality and account for individual differences among older adults, standardized procedures were implemented: questionnaires were distributed by the researchers, and participants were guided to fill them out on-site. Among them, the assessment of cognitive function was conducted by the researchers following the standardized MoCA testing process; for other self-assessment scales, participants who could read independently filled them out themselves, and the researchers provided real-time answers while they were present; for those who could not independently and smoothly fill out the questionnaire due to educational limitations, such as difficulty in reading, or difficulty in understanding some questionnaire items, researchers who had received unified training followed the neutral principle, read the questionnaire items item by item to them, and explained abstract concepts in simple and understandable language, and recorded the participants‘ clear responses. Researchers did not suggest, interpret, or modify participants' responses.

During the data review stage, questionnaires were excluded if more than 10% of items were unanswered, or if the core variables, such as MoCA cognitive assessment scores, were missing. Based on this standard, 5 questionnaires were excluded, primarily due to participant fatigue or withdrawal midway through the assessment. For the 340 questionnaires that were finally included in the statistical analysis, based on the on-site completeness verification procedure implemented during the face-to-face investigation stage, there were no missing values in the final data set. The effective recovery rate of the questionnaires was 98.6%.

### 2.6 Data analysis

Data analysis was conducted using SPSS 27.0 and AMOS 24.0 statistical software. Normality was assessed using the Kolmogorov–Smirnov test and Q–Q plots. All continuous variables met the approximate normal distribution criteria. The social demographic information and cognitive function of the participants were described using frequency, percentage, mean, and standard deviation. T-tests (such as gender, residential area, and physical exercise status) or analysis of variance (such as age, marital status, and educational background) were used to compare the demographic characteristics of the participants. Pearson correlation analysis was applied to explore the relationships among social alienation, ruminative thinking, self-neglect, and cognitive function. Additionally, this study employed confirmatory factor analysis (CFA) with a single-factor model to assess common method bias. The influence of the bias was determined by loading all theoretical dimensions of the scales into a single latent factor and comparing the fit differences with the original measurement model. Before constructing the structural equation model (SEM), this study first evaluated the multicollinearity among the main constructs using variance inflation factor (VIF) and tolerance (Tolerance). At the same time, the measurement model was evaluated using confirmatory factor analysis, and the convergent validity of each scale was measured using combined reliability (CR) and average variance extracted (AVE). Furthermore, a more rigorous chi-square difference test was used to examine the discriminant validity, comparing the baseline four-factor model with the nested model incorporating highly correlated variables. Furthermore, multi-group confirmatory factor analysis (MGCFA) was performed to evaluate the measurement invariance of the SES scale across urban and rural subgroups. A structural equation model was constructed using AMOS 24.0. To determine the model fit, the following indices were calculated: chi-square statistic (χ^2^), degrees of freedom (*df* ), chi-square statistic/degrees of freedom (χ^2^/*df* ), normed fit index (NFI), comparative fit index (CFI), Tucker-Lewis index (TLI), and approximate root mean square error (RMSEA). Finally, the bootstrap method (5,000 resamples) was used to examine the mediating associations of ruminative thinking and self-neglect in the relationship between social alienation and cognitive function (two-tailed) with a significance threshold of *p* < 0.05.

To further examine whether the hypothesized ordering between self-neglect and cognitive function was uniquely supported by the data, an exploratory reversed model was additionally tested. In this model, cognitive function was specified as a predictor of self-neglect rather than self-neglect as a predictor of cognitive function, while the remaining theoretical ordering was retained. The fit of the reversed model was evaluated using the same fit indices as the hypothesized model, and bootstrap analysis with 5,000 resamples was used to examine the reversed sequential indirect association. This analysis was conducted as an exploratory sensitivity analysis and was not intended to establish causal direction because of the cross-sectional design.

### 2.7 Ethics

This study was approved by the Ethics Committee of Anhui Medical University (approval number 81250965), and was conducted in accordance with the principles of the Declaration of Helsinki. Before formal participation, the researchers briefly confirmed whether potential participants could understand the study procedures and express their willingness to participate. Individuals with a previous diagnosis of dementia or severe neurocognitive disorder, or those who were unable to communicate their own responses, were not enrolled. The participants were informed of the purpose and significance of the study. For those who could understand the study's purpose and express their own intentions independently, written informed consent was obtained from them. For those who had difficulty reading or understanding the written information, the researchers explained the research content orally in simple and understandable language. Under the condition that the participants themselves were able to understand the study information after explanation and express their willingness to participate, signed written informed consent was obtained from the participants. When necessary, legal guardians or primary caregivers were also informed and provided an accompanying signature on the informed consent form. The questionnaire was filled out anonymously, and participants could choose to withdraw at any time during the entire study. We stated in writing that the respondents' information would be kept confidential and would only be used for this study. Written informed consent was obtained from all participants.

## 3 Results

### 3.1 Test of common method bias

This study employed the confirmatory factor analysis single-factor model method to assess common method bias. We loaded a total of 20 dimension indicators, including social alienation (4 dimensions), ruminative thinking (3 dimensions), self-neglect (5 dimensions), and cognitive function (8 dimensions), onto a single latent factor to construct a single-factor model. The results showed that the fit indices of the single-factor model were poor: χ^2^/*df* = 4.711, GFI = 0.798, CFI = 0.850, TLI = 0.833, RMSEA = 0.105. In contrast, the four-factor measurement model in this study had good fit: χ^2^/*df* = 2.200, GFI = 0.906, CFI = 0.953, TLI = 0.946, RMSEA = 0.060. The significant fit difference between the single-factor model and the measurement model indicates that there is no single common factor that can explain most of the variance in the data. Therefore, there was no evidence of serious common method bias in this study.

### 3.2 Characteristics of participants

Table 1 presents the demographic characteristics of the participants. Among them, 192 were female and 148 were male. Approximately 90% of older adults were under the age of 80, and over 70% lived in urban areas. Regarding recruitment setting, 207 participants (60.9%) were recruited from hospital settings and 133 participants (39.1%) from community settings. Among the hospital-based participants, 94 were recruited from geriatric medicine outpatient clinics, 76 from geriatric medicine inpatient wards, and 37 from neurology outpatient clinics. The majority of older adults still lived with their spouses (73.8%), while the number of those choosing to live alone (11.5%) or with their children (14.1%) was almost equal. More than half of older adults had a low educational level, with only primary school education or less. More than 70% of older adults reported engaging in daily physical exercise.

**Table 1:** Characteristics of participants (*n* = 340).

Characteristic	*N* (%)	Cognitive function	*t*/F	*P*
Gender				
Male	148 (43.5)	17.28 ± 6.65	3.351	0.002
Female	192 (56.5)	14.63 ± 7.63		
Age (years)				
< 70	180 (52.9)	16.93 ± 7.43	5.087	0.007
70–79	120 (35.3)	14.73 ± 7.05		
≥80	40 (11.8)	13.78 ± 6.95		
Marital status				
Single	0 (0)	–	8.396	< 0.001
Married	307 (90.3)	16.28 ± 7.14		
Divorced	6 (1.8)	7.83 ± 8.11		
Widowed	27 (7.9)	11.89 ± 7.39		
Educational background				
Elementary school degree or below	174 (51.2)	14.39 ± 7.63	4.935	0.002
Middle school degree or below	86 (25.3)	17.00 ± 6.96		
Secondary vocational school or high school degree	58 (17.1)	16.93 ± 6.36		
Associate or Bachelor's Degree	22 (6.5)	19.00 ± 6.74		
Occupation				
Blue–Collar or White–Collar Worker	49 (14.4)	20.49 ± 5.39	7.409	< 0.001
Farmer	85 (25.0)	15.13 ± 7.27		
Unemployed or Retired	198 (58.2)	14.74 ± 7.36		
Public institution	2 (0.6)	16.50 ± 13.44		
Others	6 (1.8)	20.67 ± 2.94		
Current residence				
Rural	90 (26.5)	9.54 ± 5.96	−10.940	< 0.001
Town or City	250 (73.5)	18.03 ± 6.43		
Living arrangements				
Living alone	39 (11.5)	11.31 ± 7.27	7.405	< 0.001
Living with spouse	251 (73.8)	16.76 ± 6.99		
Living with children	48 (14.1)	14.44 ± 7.42		
Nursing home resident	2 (0.6)	12.50 ± 16.26		
Weekly frequency of engagement in social activities (times)				
< 1	228 (67.1)	14.20 ± 7.41	18.090	< 0.001
1–2	52 (15.3)	18.48 ± 6.55		
≥3	60 (17.6)	19.47 ± 5.54		
Daily physical exercise				
Yes	250 (73.5)	17.21 ± 6.85	6.310	< 0.001
No	90 (26.5)	11.82 ± 7.20		
Cognitive status				
Normal	79 (23.2)	23.51 ± 2.17	–	–
Cognitive impairment	261 (76.8)	13.44 ± 6.71		
Recruitment setting				
Hospital	207 (60.9)	14.36 ± 7.28	–	–
Community	133 (39.1)	17.99 ± 6.89		

Cognitive status and recruitment setting were reported for descriptive purposes only and were not included in the univariate comparison.

Furthermore, based on the education level correction boundaries determined in the previous section, the participants in this study (340 individuals) were classified. Among them, 261 individuals (76.8%) were identified as having cognitive dysfunction, while the remaining 79 participants (23.2%) had normal cognitive function. When stratified by recruitment setting, 175 of the 207 hospital-based participants (84.5%) were classified as having cognitive dysfunction, whereas 86 of the 133 community-based participants (64.7%) were classified as having cognitive dysfunction.

### 3.3 Means, standard deviations and correlations among variables

Table 2 presents the average values, standard deviations, and Pearson correlation coefficients of social alienation, ruminative thinking, self-neglect, and cognitive function among older adult participants in this study. The results show that the cognitive function score of older adults was 15.78 ± 7.33, indicating a high prevalence of cognitive impairment within this cohort. The correlations indicate that social alienation (*r* = −0.749, *p* < 0.01), ruminative thinking (*r* = −0.724, *p* < 0.01), and self-neglect (*r* = −0.742, *p* < 0.01) were all strongly and negatively correlated with cognitive function.

**Table 2:** Means, Standard Deviations (SD), and Correlations Among Variables.

Variable	M (SD)	1	2	3	4
1.Social Alienation	36.41 **(**5.22)	1			
2.Ruminative Thinking	52.12 **(**10.48)	0.648^**^	1		
3.Self–Neglect	19.19 **(**5.99)	0.714^**^	0.687^**^	1	
4.Cognitive Function	15.78 **(**7.33)	−0.749^**^	−0.724^**^	−0.742^**^	1

** *p* < 0.01.

### 3.4 Multicollinearity test

Due to the high correlation among the variables, we conducted a further multicollinearity test. The results showed that the tolerance values of each predictor variable ranged from 0.403 to 0.477, all of which exceeded 0.1, and the VIF values ranged from 2.095 to 2.479, all of which were lower than 5. Additionally, the maximum condition index of the model was 17.391, which was lower than 30. This indicates that there is no serious multicollinearity among the variables in this study.

### 3.5 Evaluation of the measurement model

#### 3.5.1 Convergent validity testing

This study evaluated the convergent validity of the measurement model through confirmatory factor analysis. The results are summarized in Table 3. The composite reliability of each latent variable ranged from 0.843 to 0.897, all of which were higher than the recommended threshold of 0.70 (28). The AVE values were above 0.50 for most constructs, indicating generally acceptable convergent validity. However, the AVE for cognitive function was 0.482, which was marginally below the conventional threshold of 0.50. Because the CR value for cognitive function was 0.879, exceeding the recommended threshold of 0.70, its convergent validity was still considered acceptable according to the criterion proposed by Fornell and Larcker (29). Nevertheless, this borderline AVE value suggests that the convergent validity of the cognitive function construct should be interpreted with caution.

**Table 3:** Assessment of the reliability and convergent validity of measurement models.

Constructs	CR	AVE
Social alienation	0.897	0.687
Ruminative thinking	0.885	0.722
Self–neglect	0.843	0.520
Cognitive function	0.879	0.482

#### 3.5.2 Discriminant validity test

In terms of validity verification, given the high correlation among social psychological factors, abnormal behaviors, and cognitive decline symptoms in the older adult population, this study further employed a more rigorous Chi-square difference test to confirm the discriminant validity between latent variables (30).

We compared the baseline four-factor model (χ^2^ = 360.850, *df* = 164) with a restricted model. In this restricted model, the pair of variables with the strongest correlation, self-neglect and cognitive function (r_latent_ = −0.849), were combined into a single factor. This disattenuated correlation coefficient was estimated within the CFA framework. By accounting for measurement error, latent correlations typically demonstrate higher magnitudes than the observed Pearson correlations calculated from raw composite scores. The analysis results indicated that the fit of the combined model significantly deteriorated (χ^2^ = 447.491, *df* = 167), and the difference in the chi-square values between the two models reached a significant level (Δχ^2^ = 86.641, Δ*df* = 3, *p* < 0.001). This suggests that treating the two constructs as a single latent variable would significantly reduce the model fit, supporting their statistical distinction. Additionally, according to Kline's suggestion (31), if the absolute value of the correlation coefficient between constructs is less than 0.85, the discriminant validity is acceptable. The highest latent correlation coefficient in this study was 0.849, which was just below this boundary. In conclusion, the measurement model of this study showed acceptable discriminant validity. Nevertheless, because the latent correlation between self-neglect and cognitive function was close to the conventional boundary, the discriminant validity between these two constructs should be interpreted with caution.

#### 3.5.3 Cross-regional measurement equivalence test

Since self-neglect is the core mediating variable of this study, in order to further verify the construct validity and measurement equivalence of this scale among people in different living environments, this study used the urban-rural background as the grouping variable and conducted a multigroup confirmatory factor analysis.

This study conducted a chi-square difference test between the unrestricted baseline model and the model with equal factor loadings across groups. The unrestricted baseline model fitted well (χ^2^/*df* = 2.155, CFI = 0.906, IFI = 0.909, RMSEA = 0.058). After setting the factor loadings of the two groups as equal, the results showed that the model fit did not show a significant decline. The Δχ^2^ = 12.998, Δ*df* =13, *p* = 0.448, ΔNFI = 0.008, ΔIFI = 0.001, ΔCFI = 0.000, ΔRMSEA=-0.002. These results statistically supported factor loading equivalence of the scale across urban and rural older adult populations. This indicates that the measurement structure and item meanings of the scale remain highly consistent among different background groups.

### 3.6 Structural equation modeling results

The mediating model consists of one independent variable, one dependent variable, and two mediating variables. In this study, ruminative thinking and self-neglect were taken as the mediating variables. Through chain mediation analysis, the underlying associations between social alienation and cognitive function were explored. To partially account for potential demographic confounding, gender, age, and education level were included as control variables in the analysis.

**Figure 2:**
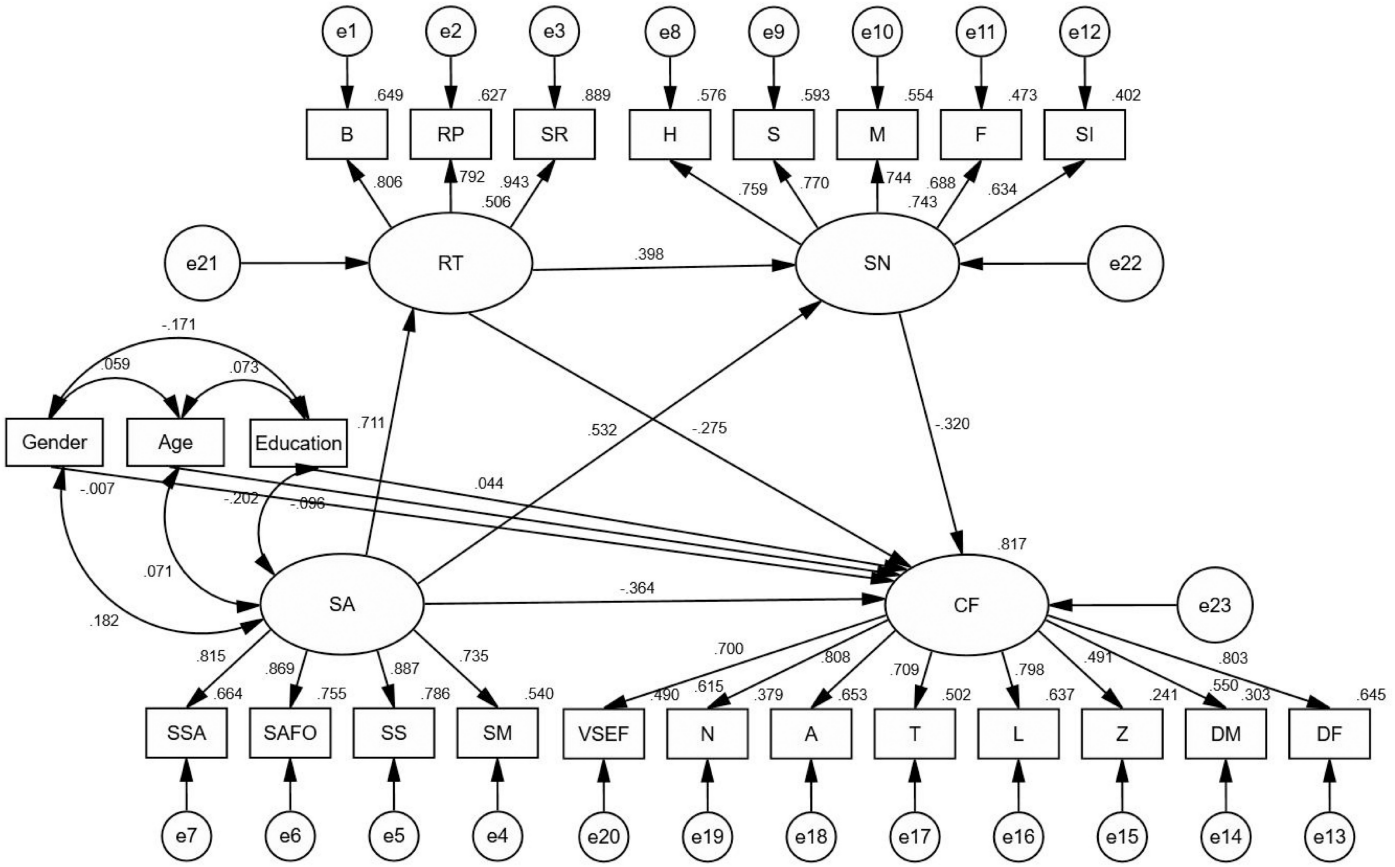
Standardized estimates of the hypothesized mediation model. SA, Social Alienation; RT, Ruminative Thinking; SN, Self-Neglect; CF, Cognitive Function; SSA, Sense of Self-Alienation; SAFO, Sense of Alienation From Others; SS, Sense of Suspicion; SM, Sense of Meaninglessness; B, Brooding; RP, Reflective Pondering; SR, Symptom Rumination; H, Healthcare; S, Sanitation; M, Emotion; F, Safety; SI, Social Interaction; VSEF, Visual-Spatial and Executive Functions; N, Naming; A, Attention; T, Calculation; L, Language; Z, Abstraction; DM, Delayed Memory; DF, Orientation.

First, the goodness-of-fit of the constructed structural equation model was tested. The model is shown in Figure 2. Model fit was evaluated using multiple fit indices. The final model demonstrated an acceptable-to-good overall fit to the data (χ^2^=460.930, *df* =218, χ^2^/*df* =2.114, CFI=0.943 TLI=0.934, NFI=0.899, RMSEA=0.057). Although the NFI value was marginally below the commonly used 0.90 threshold and should therefore be interpreted as borderline rather than unequivocally good, the remaining fit indices met commonly recommended criteria. Because model fit should be evaluated using multiple indices rather than relying on a single index, the χ^2^/*df* , CFI, TLI, and RMSEA values jointly supported the overall adequacy of the model fit. As illustrated in Figure 2, all structural paths were statistically significant (detailed path coefficients are available in Supplementary Table S1).

Table 4 presents the mediating associations of ruminative thinking and self-neglect in the relationship between social alienation and cognitive function among older adults. The results show that the confidence intervals of all paths do not include 0, indicating that all tested associations in this model were statistically significant. The estimated total association (β = −0.820, 95% CI = −0.892,−0.742) and direct association (β = −0.364, 95% CI = −0.606,−0.178) between social alienation and cognitive function were statistically significant. Most importantly, further analysis indicated statistically significant indirect associations between social alienation and cognitive function through ruminative thinking (β = −0.195, 95% CI = −0.334,−0.058), through self-neglect (β = −0.170, 95% CI = −0.368,−0.040), and through the sequential association involving both ruminative thinking and self-neglect (β = −0.091, 95% CI = −0.232,−0.017) accounting for 23.78%, 20.73%, and 11.10% of the estimated total association, respectively. As shown in Table 4, the results indicated a statistically significant sequential indirect association through ruminative thinking and self-neglect (β = −0.091, 95% CI = −0.232,−0.017), which accounted for 11.10% of the estimated total association. In summary, these findings provide statistical support for the hypothesized model and the sequential indirect association through ruminative thinking and self-neglect.

**Table 4:** Path coefficients for direct, indirect, and total associations among structural variables.

Model Path	Estimate	SE	*P*	95% Confidence Interval		Proportion of total association
				Lower	Upper	
Estimated total association	−0.820	0.039	0.001	−0.892	−0.742	100%
Estimated direct association	−0.364	0.110	0.001	−0.606	−0.178	44.39%
Estimated indirect association	−0.456	0.095	0.001	−0.643	−0.269	55.61%
Estimated indirect association 1 (SA *via* RT *to* CF)	−0.195	0.073	0.007	−0.334	−0.058	23.78%
Estimated indirect association 2 (SA *via* SN *to* CF)	−0.170	0.079	0.011	−0.368	−0.040	20.73%
**Estimated indirect association 3 (SA** ***via*** **RT** ***and*** **SN** ***to*** **CF)**	-**0.091**	**0.051**	**0.010**	-**0.232**	-**0.017**	**11.10%**

The bolded text highlights the sequential indirect association through ruminative thinking and self-neglect.

### 3.7 Exploratory reversed model

Given the high correlation and close conceptual relationship between self-neglect and cognitive function, an exploratory reversed model was further tested. In this model, cognitive function was specified as a predictor of self-neglect rather than self-neglect as a predictor of cognitive function. The reversed model demonstrated acceptable-to-good fit to the data: χ^2^ = 457.096, *df* = 218, χ^2^/*df* = 2.097, NFI = 0.900, CFI = 0.944, TLI = 0.935, RMSEA = 0.057. These fit indices were comparable to those of the hypothesized model. In the reversed model, cognitive function was negatively associated with self-neglect (β = −0.422, 95% CI = −0.779,−0.072, *p* = 0.017). The reversed sequential indirect association from social alienation to self-neglect through ruminative thinking and cognitive function was also statistically significant (β = 0.120, 95% CI = 0.023, 0.283, *p* = 0.013). These findings suggest that the cross-sectional data could not uniquely establish the temporal ordering between self-neglect and cognitive function.

## 4 Discussion

Based on the stress process model, this study systematically examined the psychological and behavioral correlates statistically linking social alienation to cognitive function. The results indicate that social alienation is not only directly associated with cognitive function, but also showed statistically significant indirect associations through ruminative thinking and self-neglect. The sequential indirect association through ruminative thinking and self-neglect accounted for 11.10% of the estimated total association. This finding suggests that lower cognitive function may not be considered in relation to a single social or psychological factor alone, but rather in relation to a constellation of psychological and behavioral correlates. This study not only supports the observed associations between variables from a statistical perspective, but also further compares the relative contributions of different statistical paths, providing a more practical basis for understanding the role of social psychological and behavioral factors in cognitive function.

This study found that, despite the education level adjustment, the average cognitive function score of older adults remained relatively low, at 15.78 ± 7.33 points. This result may be partly explained by the demographic characteristics of the sample and the sampling method used in this study. On one hand, up to 51.2% of older adults in this study had an education level of primary school or below, and older adults in the high age group (≥70 years old) accounted for a large proportion. A substantial body of epidemiological evidence indicates that low education level and advanced age are important factors associated with cognitive decline (32, 33), which to some extent reflects the actual situation of weak cognitive reserve in the grassroots older adult population. Although this group had not yet been diagnosed with severe neurological diseases in the acute stage, the coexistence of multiple chronic diseases, physiological stress, and potential sleep disorders suggests that cognitive impairment in this group should not be overlooked (34, 35). On the other hand, this study adopted a convenience sampling method, and the samples were simultaneously from the community and hospitals. This may introduce selection bias. In the hospital settings, participants were mainly recruited from geriatric medicine outpatient clinics and inpatient wards, with a smaller proportion recruited from neurology outpatient clinics. Therefore, the inclusion of clinical samples may be associated with an overrepresentation of older adults with poor physical health, multiple chronic disease burdens, age-related vulnerability, or cognitive and neurological health concerns in the total sample. Studies have shown that there is a certain relationship between physical function impairment and cognitive decline (36), so this sampling characteristic may be associated with the lower overall cognitive level observed in the sample. Consistent with this interpretation, the proportion of cognitive dysfunction was higher among hospital-based participants than among community-based participants. Indeed, this also limits the application of the conclusions of this study to older adults with high cognitive reserve. However, from the perspective of public health practice, this sample characteristic still makes the findings clinically relevant for cognitively vulnerable older adults. This suggests that in the group with weak cognitive reserve and at high risk of decline, social alienation may be understood as a chronic psychological stress-related condition, and may co-occur with persistent ruminative thinking and self-neglect behaviors, which were associated with poorer cognitive function in this study. From a clinical perspective, this suggests that for the grassroots high-risk population with fragile neurocognitive reserve, the focus of clinical care and public health services may need to extend beyond symptom management at the terminal stage to include community-based screening, which may help identify individuals who require further cognitive assessment and supportive services. The results of this study may provide preliminary empirical evidence for grassroots medical institutions to refine screening strategies and to consider early psychological assessment for older adults with low cognitive reserve. Future studies should consider more balanced recruitment across different care settings, including both community-based and hospital-based settings, to further reduce potential selection bias and improve sample representativeness.

The exploratory reversed model provided additional nuance to the interpretation of the relationship between self-neglect and cognitive function. Although the hypothesized model was theoretically informed by the stress process model, the reversed model also showed comparable statistical support, and lower cognitive function was associated with greater self-neglect. This finding does not invalidate the hypothesized model, but suggests that the association between self-neglect and cognitive function may not be adequately explained by a strictly unidirectional interpretation. Therefore, self-neglect should be interpreted as a behavioral correlate associated with poorer cognitive function, while also potentially reflecting behavioral manifestations of lower cognitive performance. In community and primary care settings, observable self-neglect behaviors may help identify older adults who require not only behavioral support, but also further cognitive screening, family involvement, and continuous follow-up care.

### 4.1 The direct association between social alienation and cognitive function

Our research has found that social alienation is significantly and negatively associated with cognitive function in older adults. These findings support Hypothesis 1, which is consistent with the research results of Huang et al. (37). Cognitive reserve theory suggests that rich social interactions may provide cognitively stimulating experiences, including dialogue, information processing, and maintenance of social roles, which are relevant to the functional reserve of the brain (38, 39). Evidence from a systematic review and meta-analysis suggests that cognitive stimulation may improve general cognitive functioning and several specific cognitive domains in older adults (40). Older adults reporting higher levels of social alienation may have fewer social contacts, less frequent cognitively stimulating interactions, and lower levels of social input that are relevant to cognitive functioning. Reduced external stimulation may represent one possible contextual correlate of poorer cognitive performance (41). This result provides preliminary empirical support for the importance of maintaining social activities for older adults, highlighting the potential relevance of maintaining substantive social connections for cognitive health in the context of an aging society. Moreover, it also suggests that the reduction in social participation may be an important marker associated with poorer cognitive function, and may have potential value for risk identification in community health management.

### 4.2 The indirect association through ruminative thinking

Further analysis indicates that ruminative thinking showed a partial mediating association between social alienation and cognitive function, supporting Hypothesis 2. It is notable that the estimated mediating association involving ruminative thinking was the largest among the tested indirect associations. This result suggests that cognitive coping styles may play an important role among factors associated with cognitive function in older adults. When older adults report higher levels of social alienation due to a shrinking social network and reduced interpersonal communication, they may have less external social stimulation and emotional support in their daily lives. This less supportive social context may be accompanied by a more persistent and repetitive negative thinking pattern. Ruminative thinking has been associated with greater cognitive resource demands and lower motivation to engage in beneficial cognitive activities and social interactions (42, 43). Previous studies have also shown that older adults who report higher levels of ruminative thinking may have difficulty shifting attention from internal negative emotions to the external social environment (44). Therefore, ruminative thinking may be considered a psychological correlate that links social alienation with poorer cognitive function.

### 4.3 The indirect association through self-neglect

This study also found a statistically significant indirect association through self-neglect in the relationship between social alienation and cognitive function in older adults, providing statistical support for Hypothesis 3 within the hypothesized model. However, given the close empirical and conceptual relationship between self-neglect and cognitive function, this finding should be interpreted cautiously. Self-neglect should not be regarded as a confirmed antecedent of poorer cognitive function in the present cross-sectional data, but rather as a behavioral correlate closely related to cognitive vulnerability. Social alienation often accompanies the lack of necessary attention and supervision from family members, friends, and society in the daily lives of older adults. This lack of social support may correspond with lower levels of basic self-care (45–47). Specifically, this is manifested in multiple aspects such as insufficient personal hygiene maintenance, reduced utilization of medical services, decreased medication compliance, and reduced safety of the living environment (48). These behavioral difficulties may be associated with poorer physical health and may also coincide with fewer opportunities or reduced ability to engage in cognitively beneficial activities (49). The theory of social support suggests that continuous social interaction may provide emotional support and external resources for maintaining healthy behaviors. Therefore, in efforts to maintain cognitive health in older adults, their social relationships and support systems should also be considered in addition to physical health. In community and primary care settings, observable self-neglect behaviors, such as poor hygiene maintenance, reduced medical help-seeking, poor medication adherence, unsafe home conditions, or withdrawal from social interaction, may alert healthcare workers to the need for further cognitive screening and supportive management. For older adults showing these behaviors, community health workers may consider combining behavioral guidance with family support assessment, home safety evaluation, medication management, and regular follow-up.

### 4.4 The sequential indirect association through ruminative thinking and self-neglect

A key finding of this study is that ruminative thinking and self-neglect demonstrated a statistically significant sequential indirect association in the relationship between social alienation and cognitive function, providing statistical support for Hypothesis 4 within the hypothesized model. As a vulnerable group in society, older adults may experience changes in family structure, limited social support, and negative attitudes toward aging, which may be associated with higher levels of social alienation (46). A qualitative study on social alienation among older adult lung cancer patients showed that their social alienation experiences included pessimism, self-doubt, and psychological burden (50). Therefore, higher levels of social alienation may be accompanied by lower perceived social identity and self-worth among older adults, which is linked to a persistent ruminative thinking pattern. This thinking pattern not only shows repeated thinking about negative emotions, but more importantly, may reflect a persistent pattern of negative cognition that is associated with greater psychological burden. This psychological burden may be associated with lower self-efficacy, more negative expectations regarding self-care behaviors, and poorer executive functioning. This state of psychological and behavioral imbalance may be closely associated with self-neglect among older adults (51–53). According to the self-regulation theory (54), poorer planning and decision-making abilities may be statistically associated with self-neglect and lower levels of advanced cognitive functioning in older adults. Based on the above content, cognitive function in older adults is associated with multiple factors. In older adult health services, future intervention research may consider a comprehensive approach that incorporates social support, psychological support, and behavioral guidance.

### 4.5 Limitations

This study still has certain limitations. Firstly, this study adopted a cross-sectional design, and all variables were measured at the same time point, which limited the establishment of the temporal sequence and causal inference. Although this study was guided by the stress process model and specified a theoretical directional framework among social alienation, ruminative thinking, self-neglect, and cognitive function, the observed results should be interpreted as statistical associations rather than confirmed causal pathways. In particular, the exploratory reversed model also showed statistical support, suggesting that the temporal ordering between self-neglect and cognitive function could not be definitively established in this study. Therefore, the association between self-neglect and cognitive function should be interpreted cautiously and may reflect a closely interrelated rather than strictly unidirectional relationship. Future studies should adopt longitudinal designs, cross-lagged panel models, or intervention studies to further clarify the temporal sequence and potential causal relationships among these variables. Secondly, due to the convenience sampling method, the sample of this study mainly came from a specific area and has significant demographic characteristics such as a low level of education and a high proportion of people with cognitive impairment. This to some extent limits the generalizability of the conclusions of this study to older adult groups with high cognitive reserves or normal cognitive function. Future research should fully consider cultural and socio-economic background factors and include diverse older adult populations from multiple regions. Thirdly, although this study controlled major demographic variables in the model, it did not comprehensively assess key clinical and psychological confounding factors such as comorbidity index, depressive symptoms, medication use, and daily functional status. Since these factors are closely related to social alienation and are also associated with cognitive decline, their absence may introduce unmeasured confounding bias. Therefore, the estimated direct and indirect associations observed in this study may be affected to some extent. Future research should quantify physical health-related data and incorporate these variables as covariates into the model to further assess the robustness of the observed mediating associations. Fourthly, the high proportion of cognitive impairment in the sample may pose potential challenges to the understanding and response quality of self-assessment scales. Although the study strictly followed standardized auxiliary procedures during the investigation and the statistical analysis confirmed the good reliability of the scales, it cannot fully rule out the reporting bias related to cognitive impairment. Future research can consider introducing objective biological indicators for multi-source cross-validation to further enhance the accuracy of social psychological assessment. Fifthly, this study mainly relied on participants' self-reported data and adopted a cross-sectional research design, which still has the potential risk of common method bias theoretically. Although common method bias was assessed using a confirmatory factor analysis single-factor model and the results showed that the bias was within an acceptable range, this remains a limitation of this study. In future research, more robust statistical control methods, such as latent variable construct method, should be considered to further enhance the reliability of the research conclusions. Sixthly, although the measurement model showed generally acceptable psychometric performance, several borderline indicators should be acknowledged. Specifically, the latent correlation between self-neglect and cognitive function was high (r_latent_ = −0.849), approaching the conventional boundary for discriminant validity, and the AVE for cognitive function was 0.482, marginally below the conventional threshold of 0.50. Although the chi-square difference test supported the statistical distinction between self-neglect and cognitive function, and the CR value supported the acceptable convergent validity of the cognitive function construct, these findings indicate that the measurement model still has certain psychometric limitations. More importantly, the close empirical relationship between self-neglect and cognitive function suggests that some self-neglect behaviors may overlap with functional or behavioral manifestations of lower cognitive performance. Future studies should further validate this model using independent samples, more diverse cognitive assessment tools, and multi-source data, such as caregiver reports, clinical assessments, or objective functional indicators.

### 4.6 Impact

This study provides preliminary insights that may inform the development of multi-level public health strategies to support cognitive health in older adults. First, at the primary healthcare practice level, healthcare systems may consider improving early screening mechanisms for cognitive disorders in community settings. During routine follow-ups and health management, community healthcare workers may consider self-neglect behaviors and ruminative thinking as psychosocial and behavioral markers associated with poorer cognitive function, in addition to physiological indicators. The exploratory reversed model further suggests that self-neglect behaviors may serve as warning signals for cognitive vulnerability. Therefore, during routine follow-ups, older adults presenting with self-neglect behaviors may require not only behavioral guidance and social support, but also timely cognitive assessment and continuous follow-up management. Strengthening relevant professional training may help grassroots healthcare workers identify older adults who require further cognitive assessment and supportive management. Second, at the community intervention level, future programs may consider comprehensive strategies covering social, psychological and behavioral dimensions. Community-based activities, such as intergenerational interaction and volunteer activities, may help increase social participation and potentially reduce feelings of social alienation among older adults. In addition, mindfulness-based stress reduction or cognitive-behavioral intervention programs may be considered to address negative cognitive patterns such as ruminative thinking. For older adults with weak social support networks or reduced self-care ability, targeted life care and health guidance may be provided to support their physical and cognitive health. For older adults living alone or lacking family members' companionship, community-based services may focus on promoting social participation by developing tailored participation plans, encouraging them to join mutual-support groups, and integrating social interaction into daily health management. These efforts may help reduce feelings of social alienation and strengthen social resources related to cognitive health among older adults. Finally, at the public policy level, health administrative departments may consider incorporating psychological status assessment and self-care ability assessment into basic public health services for older adults and community-based health records. Such efforts may contribute to the development of primary prevention-oriented services for cognitive disorders in older adults and support the provision of personalized and continuous care through multidisciplinary collaboration. This public health management model may have potential value for developing prevention-oriented cognitive health services and may provide policy references for addressing neurocognitive health challenges in the context of population aging.

## 5 Conclusion

In conclusion, based on the stress process model, this study identified a theory-informed statistical pattern involving the mediating roles of ruminative thinking and self-neglect in the association between social alienation and cognitive function among older adults in China. The exploratory reversed model further suggested that the temporal ordering between self-neglect and cognitive function could not be definitively established. The findings suggest that cognitive health promotion among older adults may benefit from comprehensive strategies that integrate social support, psychological support, behavioral guidance, and cognitive risk screening. These findings provide preliminary empirical evidence that may inform targeted public health strategies and support the transition from treatment-oriented approaches to preventive and health-promoting models of older adult care. Future longitudinal and intervention studies are needed to examine the temporal sequence and potential causal relationships among these variables.

## Data Availability

The original contributions presented in the study are included in the article/Supplementary material, further inquiries can be directed to the corresponding author.
