# Supplementary material for: Social alienation and cognitive function among older adults: the mediating roles of ruminative thinking and self-neglect
Source: Front Public Health. 2026 Jun 11;14:1820738. doi: 10.3389/fpubh.2026.1820738 (PMC13293907; doi:10.3389/fpubh.2026.1820738)
Supplement: Supplementary file 1 [file Table_1.docx]

**TABLE S1** Path coefficients of structural variables.

| **Variable links** | **Estimate** | **S.E.** | **C.R.** | ***P*** |
| --- | --- | --- | --- | --- |
| SA *to* RT | 0.711 | 0.132 | 11.074 | <0.001 |
| SA *to* SN | 0.526 | 0.073 | 7.534 | <0.001 |
| SA *to* CF | -0.364 | 0.094 | -4.899 | <0.001 |
| RT *to* SN | 0.398 | 0.033 | 6.135 | <0.001 |
| RT *to* CF | -0.275 | 0.038 | -4.376 | <0.001 |
| SN *to* CF | -0.320 | 0.106 | -3.660 | <0.001 |

*Note:* SA,Social alienation. RT, Ruminative thinking. SN, Self-neglect. CF, Cognitive function.
